# Complementary and Alternative Therapies for Functional Gastrointestinal Diseases 2016

**DOI:** 10.1155/2017/2089165

**Published:** 2017-08-07

**Authors:** Jiande D. Z. Chen, Jieyun Yin, Xiaohua Hou, Toku Takahashi

**Affiliations:** ^1^Division of Gastroenterology and Hepatology, Department of Medicine, Johns Hopkins University School of Medicine, Baltimore, MD, USA; ^2^Department of Medicine, Huazhong University of Science and Technology, Union Hospital, Wuhan, China; ^3^Department of Surgery, Medical College of Wisconsin, Milwaukee, WI, USA

Functional gastrointestinal diseases (FGID) refer to gastrointestinal diseases without known organic causes. They are well defined in a number of Rome criteria [1]. Because of unknown/unclear causes of the diseases, each of them is defined based on symptoms and presentations of symptoms. In the esophagus, common FGID include swallowing disorders, such as functional dysphasia, reflux diseases, such as gastrointestinal reflux, and hypersensitive esophagus, such as noncardiac chest pain. In the stomach, functional dyspepsia and gastroparesis are two major functional disorders. Abdominal pain with unclear causes or functional abdominal pain is also one common problem in FGID. FGID in the intestine include intestinal pseudo-obstruction, postoperative ileus, and irritable bowel syndrome. Diarrhea and constipation may involve both colon and rectum. FGID are very common in general population and account for 40% or more of patients seen at gastroenterology clinics.

Although the prevalence of FGID is high in general population, there have been limited treatment options since the pathophysiologies of various FGID are unclear. Currently, most treatments are given to relieve symptoms rather than underlying causes of the disease. Therefore, patients are not satisfied with conventional medical therapies and commonly seek alternative and complementary intervention. Based on various surveys, the use of alternative and complementary medicine ranges from 5% up to 70% in general population and 40% in pediatric patients, 50% in patients with inflammatory bowel diseases, and more than 50% in patients with irritable bowel syndrome [2–6].

Acupuncture, herbal medicine, and behavioral therapies are common alternative and complementary methods used for treating FGID and other diseases. Acupuncture is one of major Traditional Chinese Medicine (TCM) methods that has been practiced for several thousand years. It has been successful in treating various gastrointestinal diseases, especially FGID [7]. While acupuncture is widely used in clinical practice, academic research has adopted a method of electroacupuncture. In the initial method of electroacupuncture, electrical current was applied to generate local muscle contractions at the acupuncture point to mimic manual manipulation of acupuncture needles. The purpose of such an electroacupuncture method is to perform acupuncture in a uniform way. Although this may not be ideal for clinical practice that requires individualized treatment and modification of manipulation of acupuncture based on patient's reaction, such as “de qi,” it does serve the purpose of a uniform treatment. In recent years, the concept of neuromodulation (typically referring to electrical nerve stimulation therapy) has been adopted to electroacupuncture; that is, acupuncturists, probably mostly researchers, have been paying attention to stimulation parameters and outcome measurements similar to neuromodulation. This is expected to greatly enhance the effectiveness of acupuncture in various applications and make it a more viable therapy. Most recently, another novel method of electroacupuncture has been introduced: a method called transcutaneous electroacupuncture or transcutaneous electrical acustimulation (TEA) [8–12]. In this method, acupuncture needles are replaced with surface electrodes and electrical stimulation is performed via surface electrodes placed at the acupuncture points. While the replacement of acupuncture needles with surface electrodes might reduce the penetration depth of electrical current, it brings the treatment from a doctor's office to patient's home, allowing more frequent administration of the therapy. In this issue, such a method was applied to treat patients with refractory gastroesophageal reflux (L. Meng et al.).

While acupuncture or electroacupuncture gains popularity in Western countries, there are regulatory hurdles for the use of herbal medicine in Western countries and therefore most of clinical practice and research have been limited in Eastern counties. Two most commonly used methods in herbal medicine include the use of well-established formulas, such as Si-Mo-tang, Liu-Jun-Zi-Tang, Daikenchuto, and the use of combination of various herbals [6]. In addition to continuing to explore clinical efficacies, recent research in herbal medicine has been focused on its biological and molecular mechanisms, such as those included in this special issue (X. Ma et al., Z. Wu et al., and Y. Lu et al.).

Behavioral therapies are also commonly used in the treatment of FGID, such as meditation, relaxation exercise, and biofeedback training. While mechanisms involved in the ameliorating effects of behavioral therapies are largely unknown, most of relaxation therapies are designed to suppress sympathetic overactivity and balance sympathetic and parasympathetic activities. Sympathetic overactivity and/or sympathovagal imbalance has been reported in patients with FGID, such as functional dyspepsia and irritable bowel syndrome [13, 14]. Unfortunately, none of papers included in this special issue is on behavioral therapies.

A total of 9 studies are included in this special issue, ranging from the esophagus to the colon. L. Meng et al. reported a clinical study using transcutaneous electrical stimulation at acupuncture points for the treatment of refractory gastroesophageal reflux. Significant improvement in symptoms of reflux was noted, probably attributed to its enhancive effect on the pressure of the lower esophageal sphincter. In patients with refractory gastroesophageal reflux, the noninvasive and home-based TEA therapy may be added to the conventional proton pump inhibitor therapy. Two studies reported the use of herbal medicine for treating gastric diseases. In an animal study by V. Bespalov et al., a tablet of Conifer Green Needle Complex was found effective in treating precancerous gastric lesion. In an in vitro study by Z. Wu et al., a Chinese herbal medicine, Aurantii fructus immaturus flavonoid was reported to reduce contractility of pyloric circular smooth muscle in a dose-dependent manner in rats via the regulatory pathway of NO/cGMP/protein kinase G/Ca^2+^. The effectiveness of Chinese herbal medicine in the treatment of functional abdominal pain was reported in a systematic review (T. Liu et al.): it revealed that the most commonly single herbal medicine was Radix Ginseng and the most commonly used herbal formula was Si-Mo-Tang. The treatment of irritable bowel syndrome is the most common topic of this special issue with two original preclinical mechanistic studies and two meta-analyses of clinical trials. In one animal study, X. Ma et al. reported the ameliorating effect of Tong-Xie-Yao-Fang on diarrhea and cellular mechanisms in a rodent model of diarrhea-dominant irritable bowel syndrome. A meta-analysis by J.-J. Zhu et al. reported effectiveness of Chinese Herbal Medicine in improving diarrhea, global symptoms, and abdominal pain in patients with diarrhea-dominant irritable bowel syndrome. Another meta-analysis by Z. Yang et al. reported that moxibustion might be effective in treating diarrhea-dominant irritable bowel syndrome compared with pharmacological medications. However, further large, rigorously designed trials are needed due to insufficient methodological rigor in the included trials.

*Jiande D. Z. Chen* *Jieyun Yin* *Xiaohua Hou* *Toku Takahashi*
